# Xia-Gibbs Syndrome: A Rare Case Report of a Male Child and Insight into Physiotherapy Management

**DOI:** 10.7759/cureus.9622

**Published:** 2020-08-09

**Authors:** Chanan Goyal, Waqar Naqvi, Arti Sahu

**Affiliations:** 1 Physiotherapy, Datta Meghe Institute of Medical Sciences, Wardha, IND; 2 Neuroscience, Government Physiotherapy College, Raipur, IND; 3 Community Physiotherapy, Datta Meghe Institute of Medical Sciences, Wardha, IND

**Keywords:** xia-gibbs syndrome, xgs, developmental delay, neurodevelopmental treatment, sensory integration, physiotherapy, motor delay, pediatric rare diseases, paediatric physiotherapist

## Abstract

Xia-Gibbs syndrome (XGS) is a recently discovered genetic disorder. It is characterized by global developmental delay, intellectual impairment, hypotonia, and sleep abnormalities. While the current literature emphasizes on the genotype and phenotype of this rare condition, it does not provide any description of the physiotherapy management of patients with XGS. We report a case of a 27-month-old Indian male diagnosed with XGS, who presented with difficulty in sitting without support. He had dysmorphic facies, hypotonia, hyperextensible joints, mild kyphoscoliosis, and global developmental delay. His parents and an elder female sibling were clinically asymptomatic. The physiotherapy intervention was based on the principles of neurodevelopmental treatment (NDT) and sensory integration (SI). The management included facilitation of transitions, weight-bearing exercises, wheelbarrow walking, joint compressions, rib cage mobilization, multidirectional reaching, and pushing-pulling activities along with the use of equipment like Swiss ball, balance board, stability disc, trampoline, swing system, walker (rollator), and walking harness. Also, stabilizing pressure input orthosis (SPIO) for the trunk and ankle-foot orthosis (AFO) followed by supramalleolar orthosis (SMO) were used for support. Thereafter, the child was able to stand and walk without support at the age of 36 months, and walk on uneven terrain at the age of 42 months. In addition, he could negotiate stairs using handrails with mild assistance. His gross motor function measure-88 (GMFM-88) total score improved from 21% at the presentation to 66.6% following the treatment. It was observed that the NDT and SI approaches along with the use of appropriate orthoses accelerated the achievement of motor milestones in this case. To the best of our knowledge, this is the first case report of a child with XGS that emphasizes on the course of physiotherapy management for the associated motor delay.

## Introduction

In 2014, Fan Xia and Richard A. Gibbs described Xia-Gibbs syndrome (XGS) for the first time. It occurs due to heterozygous mutations in the AT-hook DNA binding motif containing 1 (AHDC1) gene on chromosome 1p36 [1]. XGS is a genetic neurodevelopmental disorder characterized by intellectual impairment, global developmental delay, hypotonia, feeding problems, distinctive facial features, and obstructive sleep apnea [1,2]. In their study published in January 2020, He et al. reported that around 100 patients with XGS have been identified worldwide [3]. According to another study published in January 2020, less than 50 patients have been reported in the literature since the first description of the disease [2]. We believe there is a significant need to add more information to the growing literature on XGS, especially in terms of care [4]. To the best of our knowledge, this is the first report that emphasizes on the course of physiotherapy in a child diagnosed with XGS.

## Case presentation

**Figure 1:**
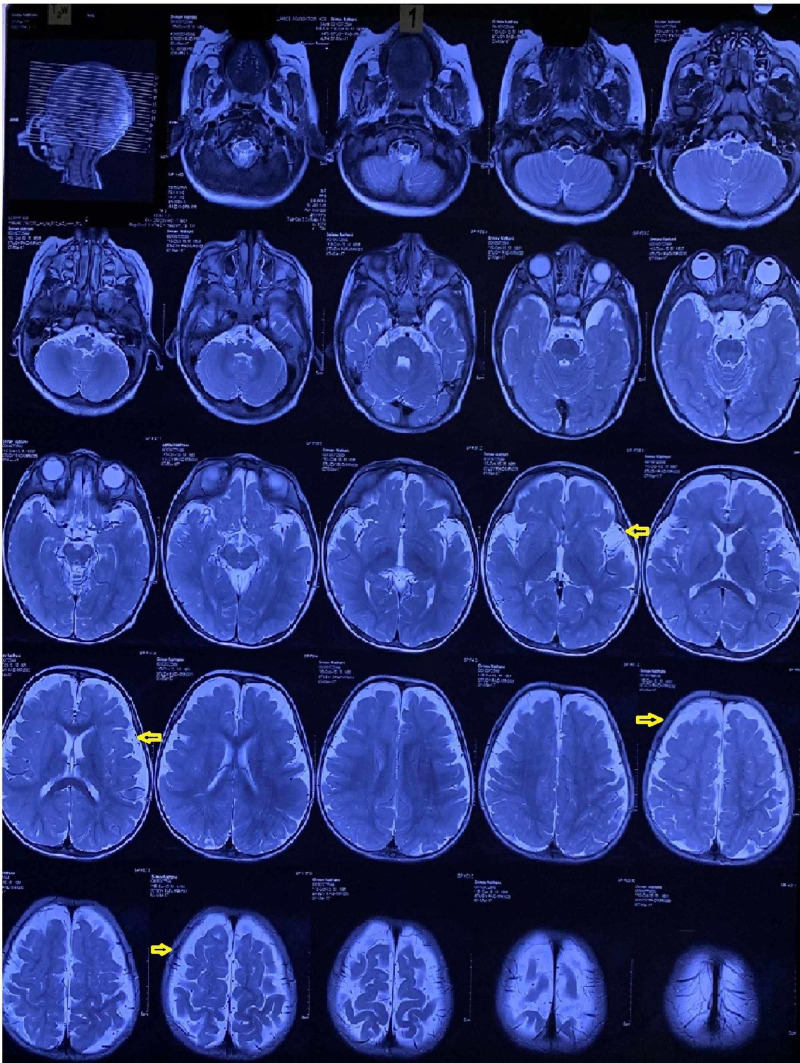
MRI of the brain (axial view) Yellow arrows depict areas showing significant cerebrocortical atrophy and dysmorphism MRI: magnetic resonance imaging

A 27-month-old male child presented to the physiotherapy department with the parents’ primary concern of his inability to sit without support. As per the mother, he had been born full-term through caesarean section, second in order of a non-consanguineous marriage. He had cried immediately after birth and had weighed 4 kilograms. His right eye had been half-open. In the first eight months of life, he had not been very active. He had been diagnosed with vitamin D deficiency, which had been managed medically. He had achieved neck control and rolling over from supine to prone at the age of 12 months, and tripod sitting at the age of 18 months. He presented with global developmental delay and failure to thrive. His parents and elder sister were clinically asymptomatic. An MRI of the brain and exome sequencing were performed, and he was diagnosed with XGS (Figure 1).

**Figure 2:**
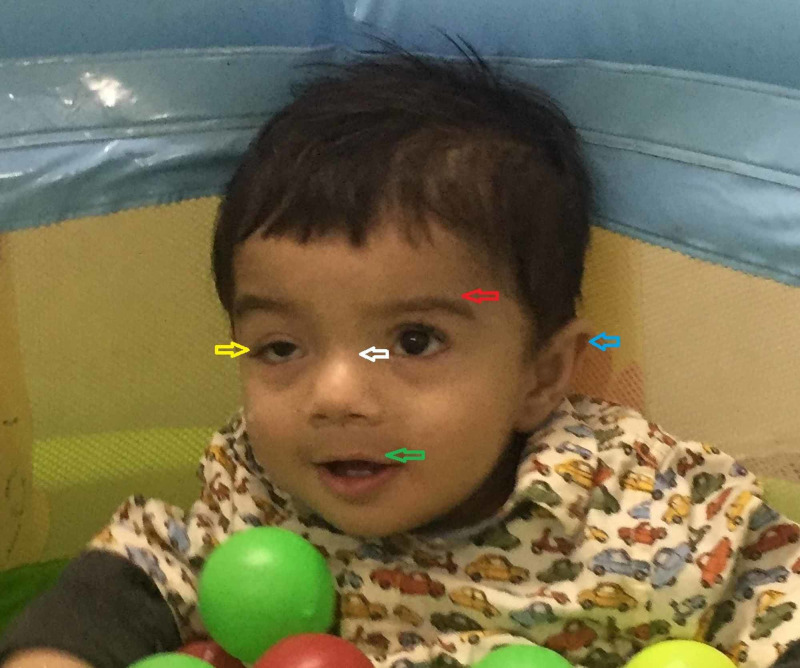
Dysmorphic facial features The image shows downward slanting palpebral fissures with partial ptosis of the right eye (yellow arrow), horizontal eyebrows (red arrow), flat nasal bridge (white arrow), thin upper lip (green arrow), and low set ears (blue arrow)

On further observation, he had dysmorphic facies including downward slanting palpebral fissures with partial ptosis of the right eye, horizontal eyebrows, flat nasal bridge, thin upper lip, full cheeks, round face, and low set ears (Figure 2).

**Figure 3:**
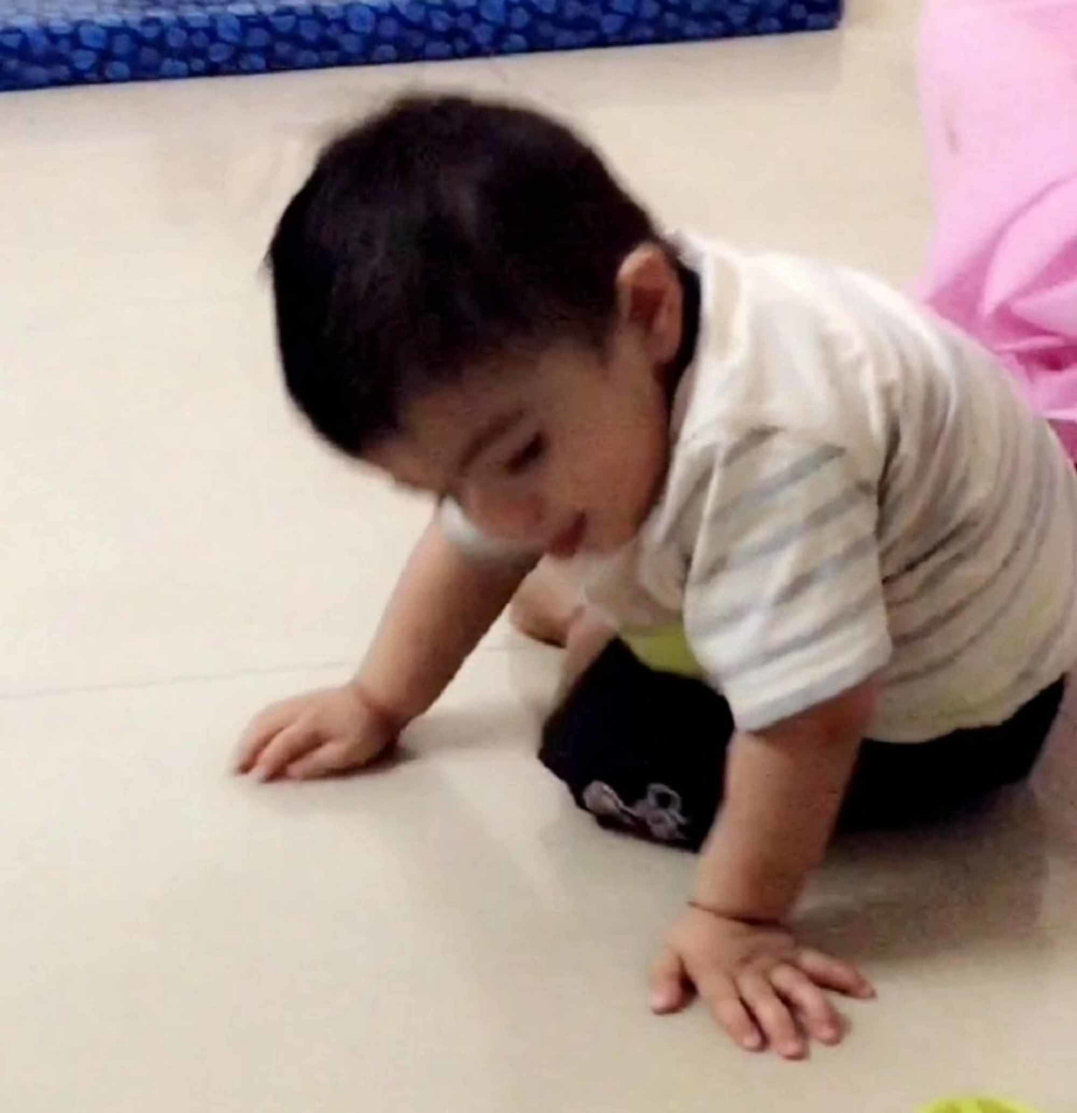
Tripod sitting Before physiotherapy intervention, the child was able to sit with support at the age of 27 months

He also had a single transverse palmar crease and wide anterior fontanelle. On examination, he had hypotonia and hyperextensible joints. During tripod sitting, he adopted forward head posture with exaggerated thoracic kyphosis (Figure 3).

He had mild right dorsolumbar scoliosis with rib cage flaring and a protuberant abdomen. He was unable to perform overhead shoulder elevation. Inferomedial border of bilateral scapulae became prominent revealing a lack of scapular stability when assisted overhead shoulder elevation was performed. He had an adequate gross grasp, but was able to hold objects for two or three seconds only, and was inclined towards throwing them. He had bilateral plano valgoid feet, which was evident while weight-bearing. Pointing and vocalization were absent. On the gross motor function measure-88 (GMFM-88) used for the evaluation of the motor skills, the child demonstrated a total score of 21% [5,6].

Therapeutic intervention

**Figure 4:**
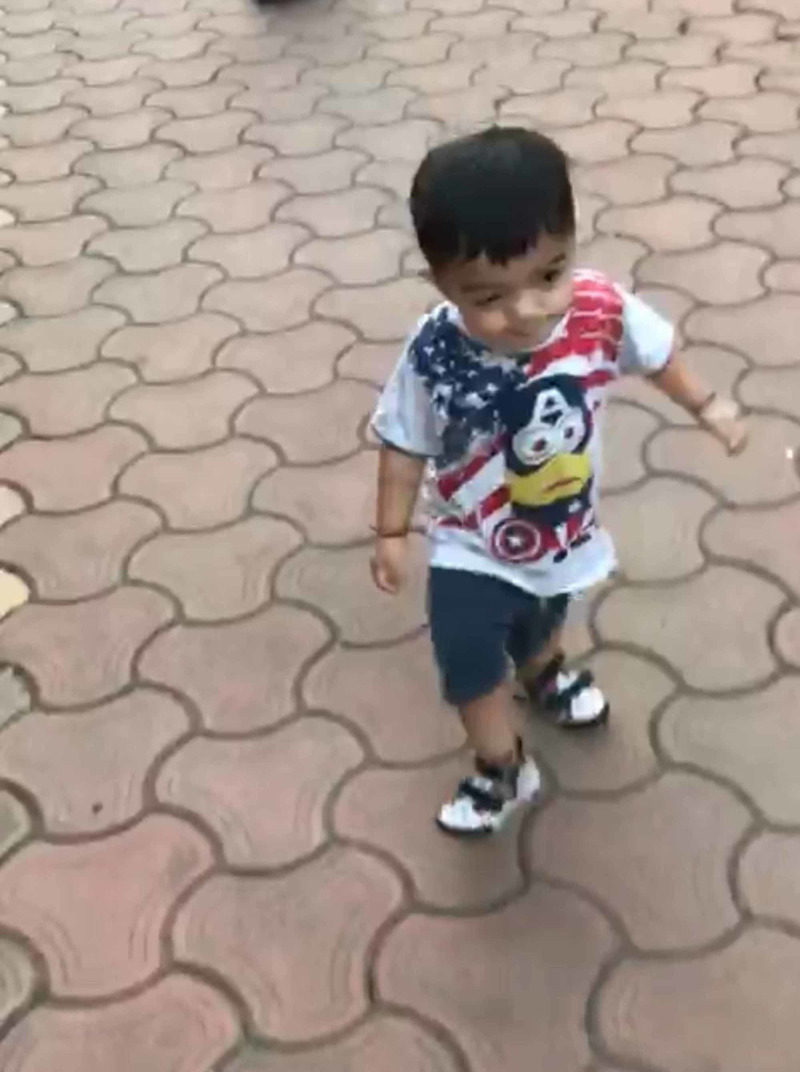
Independent walking After physiotherapy intervention, the child was able to walk without support at the age of 36 months

Physiotherapy intervention to achieve gross motor developmental milestones included neurodevelopmental treatment (NDT) and sensory integration (SI). To challenge the child’s balance, he was made to reach for objects of interest while sitting on the equilibrium board and Swiss ball. Trunk extension was practised on the Swiss ball. With trunk stabilizing pressure input orthosis (SPIO), transitions like supine to sit, sit to stand, and half kneel to stand were facilitated by appropriate placement of the therapist’s hands. Proprioceptive input was given by weight-bearing exercises and joint compressions. Vestibular input was given by the swing system. Wheelbarrow walking was practised for activating scapular muscles. Rib cage mobilization, facilitation of right side flexors of the trunk, and hanging with upper trunk support were done to manage mild scoliosis. As the child began to perform unsupported standing while wearing bilateral ankle-foot orthosis (AFO), a walker (rollator) was used as an aid for walking under close supervision. He practised standing on a balance board and stability disc with minimal support accompanied by multidirectional reaching while standing and pushing-pulling activities. He practised bouncing on a Swiss ball and trampoline with support and kicking a ball with ankle weight cuffs. Thereafter, walking was practised on a firm surface with a bilateral supramalleolar orthosis (SMO) and walking harness with minimum support. He started walking without support at the age of 36 months (Figure 4).

Walking was then practised on compliant surfaces and across the obstacle course to improve balance. Negotiating stairs by holding onto railings with moderate assistance was incorporated. Parents were educated about the home exercise program. With the regular individualized physiotherapy program, the child's total score on GMFM-88 improved to 66.6% at the age of 42 months. Table 1 shows a detailed timeline of the events.

**Table 1:** Timeline of the events MRI: magnetic resonance imaging; AHDC1: AT-hook DNA binding motif containing 1; ASD: autism spectrum disorder; GMFM-88: gross motor function measure-88; NDT: neurodevelopmental treatment; SI: sensory integration

Date	Consultation	Event(s)	Diagnosis/findings	Treatment/suggestions
15 October 2015	Obstetrician and paediatrician	Full-term caesarean section (FTCS)	Right-eye ptosis	_
7 March 2017	Radiologist	3 Tesla MRI	Significant cerebrocortical atrophy and dysmorphism	_
23 May 2017	Clinical geneticist	Exome sequencing	Gene: heterozygous variant in AHDC1 (-); location: exon 6; disease: Xia-Gibbs syndrome; inheritance: autosomal dominant	Sanger sequencing, sequencing in parents and genetic counselling
13 June 2017	Audiologist	Brainstem evoked response audiometry (BERA)	Mild hearing loss in the right ear	_
23 June 2017	Scientist	AHDC1 mutation analysis of asymptomatic parents	Father (34 years): variation absent; mother (31 years): variation present (heterozygous)	-
16 January 2018	Developmental paediatrician	Cognitive adaptive test	Developmental quotient: 44.81 (below average in motor and adaptive areas)	Physiotherapy
		Clinical linguistic and auditory milestone scale	Developmental quotient: 40.74 (significant delay in receptive and expressive language)	Speech therapy
		Diagnostic and Statistical Manual of Mental Disorders, Fifth Edition (DSM V)	Some autistic features reported, but the diagnosis of ASD deferred	Occupational therapy
		Sensory profile	Mild somatosensory and auditory stimulus processing problems	SI
22 January 2018	Paediatric physiotherapist	GMFM-88	Total score: 21%	NDT and SI
10 April 2019	Paediatric orthopaedic surgeon and radiologist	Spinal evaluation	Pectus excavatum, increased cervical lordosis, crowding of ribs at the left side, mild right dorsolumbar scoliosis	Physiotherapy
15 April 2019	Paediatric physiotherapist	GMFM-88	Total score: 66.6%	Home exercise program

## Discussion

Clinical features noticed in the child were consistent with the previous studies on XGS [2,4,7]. Single palmar crease, unilateral ptosis, and wide anterior fontanelle are unusual manifestations for the diagnosis, which were found in the child. Variation in manifestations has been noted in a previous study too [8]. A dearth of literature on the management of individuals with XGS was observed. In our case, physiotherapy intervention was planned to facilitate motor development. GMFM-88 was used for evaluation as it is considered reliable and valid for use in conditions other than cerebral palsy [5,6]. At 27 months of age, the child had difficulty in maintaining sitting without support and his GMFM-88 total score was 21%. After the course of physiotherapy along with the use of orthoses, at the age of 36 months, the child was able to stand and walk without support. At the age of 42 months, the child was able to independently walk on uneven terrain. Also, he was negotiating stairs while using handrails with mild assistance. A GMFM-88 total score of 66.6% was recorded. Improvement was observed in all five dimensions of GMFM. He could reach for objects overhead, and he was able to hold objects in hands for five minutes, unlike before. NDT and SI approaches were found to be beneficial for the child in gaining motor skills in our study. This is consistent with observations in previous studies on other neurodevelopmental disorders [9,10]. The use of SPIO helped in improving posture, which is the basis of movement [11]. AFO and SMO have positively influenced standing and walking, which is in line with findings in an earlier study [12].

## Conclusions

To conclude, the child with XGS showed an overall improvement in motor skills, suggesting that the combined NDT and SI approach along with the use of appropriate orthoses were effective in his habilitation. The ability to walk independently has contributed to his full participation in society. As this report highlights the positive impact of physiotherapy on this rare genetic disorder, further studies to explore the potential of physiotherapy in early intervention programs as well as during the life span of patients with XGS are warranted.
